# Defects Induced by High-Temperature Neutron Irradiation in 250 µm-Thick 4H-SiC p-n Junction Detector

**DOI:** 10.3390/ma18112413

**Published:** 2025-05-22

**Authors:** Alfio Samuele Mancuso, Enrico Sangregorio, Annamaria Muoio, Saverio De Luca, Matteo Hakeem Kushoro, Erik Gallo, Silvia Vanellone, Eleonora Quadrivi, Antonio Trotta, Lucia Calcagno, Francesco La Via

**Affiliations:** 1 Department of Physics and Astronomy, University of Catania, 95123 Catania, Italy; lucia.calcagno@ct.infn.it; 2 CNR-IMM, VIII Strada n 5, 95121 Catania, Italy; enrico.sangregorio@imm.cnr.it (E.S.); annnamaria.muoio@imm.cnr.it (A.M.); saverio.deluca@cnr.it (S.D.L.); 3 Department of Physics “G. Occhialini”, University of Milano-Bicocca-Piazza dell’Ateneo Nuovo 1, 20126 Milano, Italy; matteo.kushoro@unimib.it; 4 Institute for Plasma Science and Technology (ISTP), Consiglio Nazionale delle Ricerche (CNR), Via Roberto Cozzi 53, 20125 Milano, Italy; 5 Eni S.p.A, Piazzale Mattei 1, 00144 Roma, Italy; erik.gallo@eni.com (E.G.); silvia.vanellone@eni.com (S.V.); eleonora.quadrivi@eni.com (E.Q.); antonio.trotta@eni.com (A.T.)

**Keywords:** silicon carbide, p-n junction, neutron detection, high temperature, deep defect

## Abstract

The objective of the proposed work was to investigate the electrical performance of a 250 µm-thick 4H-SiC p-n junction detector after irradiation with DT neutrons (14.1 MeV energy) at high temperature (500 °C). The results showed that the current–voltage (I-V) characteristics of the unirradiated SiC detector were ideal, with an ideality factor close to 1.5. A high electron mobility (µ_n_) and built-in voltage (V_bi_) were also observed. Additionally, the leakage current remained very low in the temperature range of 298–523 K. High-temperature irradiation caused a deviation from ideal behaviour, leading to an increase in the ideality factor, decreases in the µ_n_ and V_bi_ values, and a significant rise in the leakage current. Studying the capacitance–voltage (C-V) characteristics, it was observed that neutron irradiation induced reductions in both Al-doped (p^+^-type) and N-doped (n^−^-type) 4H-SiC carrier concentrations. A comprehensive investigation of the deep defect states and impurities was carried out using deep-level transient spectroscopy (DLTS) in the temperature range of 85–750 K. In particular, high-temperature neutron irradiation influenced the behaviours of both the Z_1/2_ and EH_6/7_ traps, which were related to carbon interstitials, silicon vacancies, or anti-site pairs.

## 1. Introduction

The success of nuclear fusion as a viable energy source depends critically on the ability to generate, control, and monitor the production of tritium—the key fuel in deuterium–tritium (D–T) fusion reactions. In next-generation fusion reactors, such as SPARC, ITER, and DEMO, tritium is expected to be bred in situ through neutron–lithium interactions in the breeding blanket (BB). Monitoring the efficiency of this tritium breeding process is essential for both the scientific validation of reactor performance and for ensuring fuel self-sufficiency. However, validating BB designs under realistic operating conditions remains a major challenge due to the current unavailability of neutron sources that replicate the high-fluence volumetric environment of a tokamak. In this context, fast neutron detectors play a crucial role: they enable the quantification of neutron flux and multiplication rates and provide experimental data on the frequency and efficiency of tritium-producing reactions [1,2]. To be effective, these detectors must be compact, resistant to high radiation damage, and capable of operating at elevated temperatures within the reactor environment. Diamond detectors have demonstrated excellent time and energy resolution and have been successfully deployed in experiments at the Joint European Torus (JET), proving capable of measuring the total number of neutrons with an energy of 14 MeV produced in D-T fusion reactions [3]. Although the performance was nearly constant up to a certain temperature, Angelone et al. observed that the neutron signal decreased above 503 K [4].

For this reason, silicon carbide (SiC) is being studied as a great alternative material for neutron detectors. The physical properties of SiC allow it to perform accurate neutron flux measurements while being in environments characterized by high temperatures and irradiation. Kushoro et al. explored the high-temperature radiation hardness of SiC detectors, demonstrating their ability to withstand 14 MeV neutron irradiation without any performance degradation at 523 K [5].

Unfortunately, the presence of crystalline defects can adversely impact device performance. The crystal growth process or ion implantation are the common techniques to introduce defects in SiC [6]. While many studies have focused on the defects arising from ion implantation [7,8,9], there remains a lack of detailed data on neutron-induced defects at elevated temperatures. Specifically, high-energy neutrons are one of the most intense and permanent sources of displacement damage in SiC devices because of their non-ionizing radiation nature and considerable depth of penetration. In contrast to charged particles, neutrons merely glide through the electronic orbits of Si and C atoms with little energy expenditure, but elastic scattering of nuclei can yield primary knock-on atoms (PKAs). These PKAs set in motion displacement cascades that yield Frenkel pairs (vacancy–interstitial defects) in the crystal lattice. This phenomenon scales with the amount of damage for a given neutron fluence and the non-ionizing energy loss (NIEL), which defines the energy loss of a particle per unit path length for displacement processes in the material [10]. These defects can form stable complexes and behave as efficient recombination or trapping centres, thereby reducing carrier mobility and lifetime, the leakage current, and the overall performance of the device over time [11,12].

In this study, we investigate neutron-induced defect formation in a 250 µm-thick 4H-SiC p-n junction detector (SiC-K) developed within the Joint Research Center CNR-ENI “Ettore Majorana.” The detector was exposed to deuterium-tritium fusion neutrons with a fluence of 5 × 10^12^ n/cm^2^. The irradiation campaign extended the operational temperature up to 773 K, significantly exceeding the 523 K explored in earlier experiments at the Frascati Neutron Generator (FNG) in Italy. Its electrical performance was compared to that of an unirradiated counterpart (SiC-V). The electrical characteristics of two SiC detectors were assessed using current–voltage (I-V) and capacitance–voltage (C-V) measurements. Additionally, deep-level transient spectroscopy (DLTS) was employed to evaluate the energy activation and generation yield of defects. This paper is organized as follows: After an introduction given in Section 1, the description of the detector and experimental setup is given in Section 2. In Section 3, the main experimental results are presented and discussed, while in Section 4 the summary of the results of this work are discussed.

## 2. Materials and Methods

The 4H-SiC p-n^−^ detectors studied in this work were fabricated by the CNR Institute for Microelectronics and Microsystems. Their physical structure consisted of an active epilayer with a thickness of 250 μm grown on a commercially available <0001> 4° off-axis n^++^ 4H-SiC epitaxial wafer with a thickness of 350 μm. A 5 μm n^+^ buffer layer was introduced to mitigate defect propagation from the wafer into the active layer. A thick low-doped n-type layer (250 µm, N_D_ = 7 × 10^13^ cm^−3^) was grown using the chemical vapor deposition process in a horizontal hot-wall reactor (ASM PE106). The precursors used were ethylene as the carbon source, trichlorosilane (TCS) as the chlorinated silicon source, and nitrogen as the n-type dopant. The deposition was carried out at a pressure of 100 mbar and a temperature of 1923 K. A 0.3 μm-thick p^+^ layer with a doping concentration of 2 × 10^18^ cm^−3^ was formed through ion implantation. A silicon dioxide (SiO_2_) layer was deposited to electrically isolate the central active region of the device. Additionally, implanted p^-^ and n^+^ regions were considered to properly realize the design of a junction termination extension (JTE) and the field stop structure of the detector, respectively. The devices were produced in three different shapes, having areas of 25, 12.5, and 6.25 mm^2^. Nickel (Ni) was deposited, followed by a silicidation process to form nickel silicide in the contact regions, thereby reducing contact resistance. The unreacted nickel was then removed by etching.

**Figure 1:**
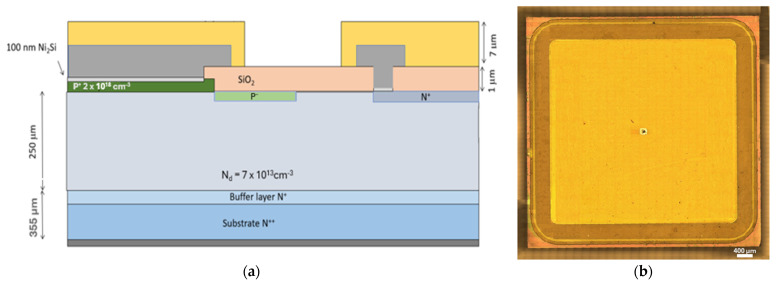
(**a**) Cross-sectional scheme of the edge structure of the 4H-SiC p-n^−^ detectors and (**b**) optical image of the finalized detectors (400 μm scale is put in the bottom right corner as a reference).

The cathode contact was made by depositing a 1 μm-thick aluminium (Al) layer. Lastly, the device was passivated using a 7 micron-thick polyimide layer. In Figure 1a the schematic of the detector structure is shown, along with a picture of the device.

The devices were tested using CVU-200-KIT bias tees, specifically designed to interface with the Keithley 2612 Source-Measure Unit (SMU) and model 4200-SCS for capacitance–voltage (C-V) measurements. Both instruments were integrated with a Nextron probe station. The devices were electrically tested in a nitrogen environment at multiple temperatures between 298 K and 523 K. At each temperature, the forward and reverse current–voltage characteristics were studied. The former was acquired by sweeping a DC bias from 0 to 5 V in steps of 25 mV; the reverse characteristics were instead acquired by sweeping a DC bias from 0 to −200 V in steps of −1 V. At each step of temperature, the capacitance–voltage characteristics were acquired by ACS Basic software sweeping a DC bias from 0 to −200 V in steps of −1 V. During all three sweeps, the compliance current was set at 10 mA. Deep-level transient spectroscopy (DLTS) measurements were carried out using a Sula Technologies double boxcar spectrometer with an exponential correlator in the temperature range of 85–750 K. The temperature of the samples was controlled using a LakeShore Cryotronics 331 Temperature Controller. At each temperature, the DLTS signals were obtained with a 2.3 V pulse signal. Pulses were applied in a reverse-bias environment of −13 V and had a temporal width of 1 ms (with delay windows from 2 to 20 ms). During the measurements, the sample chamber was kept under vacuum (at a pressure of 3.4 × 10^−5^ mbar) using a turbomolecular pump.

## 3. Results and Discussion

### 3.1. Electrical Characteristics of SiC Detectors

**Figure 2:**
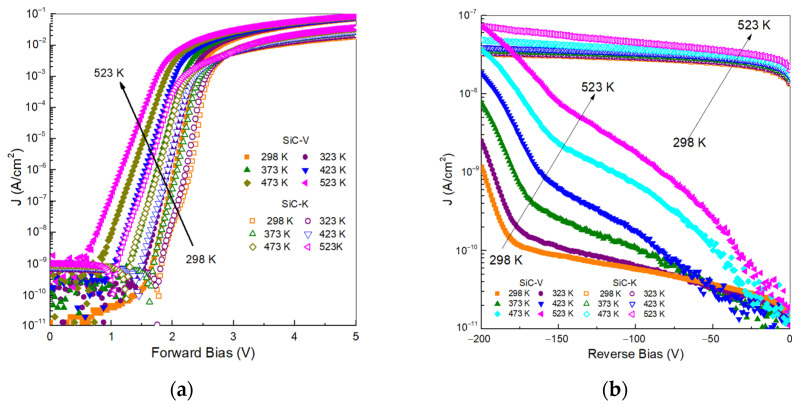
I-V characteristics. (**a**) Forward J-V and (**b**) reverse J-V curves for the unirradiated SiC-V and the irradiated SiC-K at different temperatures (298–523 K).

In a diode, several non-ideal effects cause the measured I-V curve to deviate from the exponential behaviour predicted by the ideal Shockley equation [13]. This is due to the diode resistance (R_S_), which can be approximated as the sum of the bulk material resistance, the contact resistance, and the depletion region resistance. When a sufficiently high (qV_F_ >> kT) forward bias is applied, the total current density can be approximated by the following equation [14]:

(1) $$J_{\mathrm{tot}}=J_{0}{\;e}^{\frac{{q\;(V}_{\mathrm{bi}}-V_{F}-{\mathrm{IR}}_{s})\;}{n\;k\;T}}+\frac{{\;V}_{F\;}}{R_{\mathrm{sh}}}$$

where q is the electric charge, k is the Boltzmann constant, R_sh_ is the shunt resistance, $R_{s}$ is the series resistance, and n is the ideality factor. In a 4H-SiC diode, the ideality factor can have a value between 1 and 2, depending on the specific conduction mechanism [15]. Figure 2 shows the current density-voltage (J-V) characteristics of the SiC detectors in the temperature range of 298–523 K. At room temperature, the forward current-voltage curves of the unirradiated detector (SiC-V) exhibited typical p-n junction behaviour. Figure 2a shows that the diode behaviour was dominated by the shunting resistance up to a forward bias of 1.5 V. The R_sh_ value represents the unintended low-resistance pathways that can arise from manufacturing defects, material impurities, or edge leakage currents. Beyond this point, a further increase in voltage induces only a small increase in the current density due to the onset of the series resistance [16]. The detector that was irradiated with a neutron fluence of 5 × 10^12^ n/cm^2^ (SiC-K) exhibited a similar behaviour, except for a slight increase in V_F_, possibly due to the diffusion–recombination current. In addition, the J-V characteristics showed higher series resistance due to neutron irradiation [17]. The effects of temperature on the J-V curves for both types of diodes can be compared: increasing the temperature to 523 K led to an exponential increase in the recombination–diffusion current density. On the other hand, the series resistance also increased at higher temperatures.

The ideality factor was calculated by fitting the slope of the linear region of the recombination current. At room temperature, SiC-V showed a value of 1.5, which is characteristic of an ideal diffusion–recombination process. When the temperature was increased, the value decreased to 1.1, suggesting an enhancement of the diffusion mechanism. The already irradiated detector (SiC-K) showed an ideality factor of 2.4 at room temperature, followed by a larger decrease as the temperature increased. This effect was likely due to neutron irradiation inducing defects in the SiC epilayer or in the implanted p^+^ layer, forming trapping levels within the band gap and thus inducing an increase in the ideality factor.

Figure 2b shows when the reverse bias increases above -180 V for the two detectors. For SiC-V, the leakage current density increased significantly when the bias fell below −180 V. At this reverse bias, the leakage current density was in the range of 1 to 100 nA/cm^2^ for temperatures between 298 and 523 K. The rise in leakage reverse current in SiC-V at high temperatures was a multifaceted phenomenon. It stemmed from the exponential increase in thermally generated carriers, the activation of defect states that enabled trap-assisted tunnelling, and the enhancement of thermionic emission. After neutron irradiation, the reverse current increased by almost two orders of magnitude at a reverse bias of 200 V in SiC-K, as depicted in Figure 2b. A similar behaviour was also observed by L.Y. Liu et al. in the I-V characteristics of Ni/4H-SiC Schottky detectors, having identified a detector’s leakage current one order of magnitude higher after being exposed to a neutron fluence of 2.05 × 10^13^ n/ cm^2^ [18]. The induced defects in the depletion region of their SiC detectors were generally considered to be responsible for it. Consequently, the irradiation-induced defects facilitated trap-assisted tunnelling and contributed to additional pathways for thermal generation of electron–hole pairs. They also found that performance degradation at the nickel-SiC interface caused by neutron irradiation can also influence its performance.

High-temperature operation at 773 K led to the degradation of the aluminium metallization in SiC-K, increasing the densities of electrically active defects and interface states [19]. Additionally, neutron irradiation introduced lattice defects, further affecting the material’s electrical properties. As a result, the leakage current in the SiC-K diode increased.

When external V_F_ is applied to the SiC detectors, the effective barrier potential opposing carrier movement was reduced. Specifically, as V_F_ increased, the net potential barrier decreased as (V_bI-VF_), allowing more charge carriers to cross the junction, thereby increasing the current flow through the diode. In the recombination–diffusion regime, the contribution of shunt and series resistances had a negligible effect on J_tot_ as shown in Figure 2a. Thus, Equation (1) can be approximated as follows:

(2) $$J_{\mathrm{tot}}\approx {\;J}_{0}{\;e}^{\frac{{q\;(V}_{\mathrm{bi}}-V_{F\;}-{\mathrm{IR}}_{s})\;}{n\;k\;T}},$$

Consequently, we can extrapolate V_bi_ values from Equation (2), knowing that:

(3) $$V_{\mathrm{bi}}=V_{F}+\frac{\mathrm{nk}\;T}{q}\;\ln\left(\frac{J_{\mathrm{tot}}}{J_{o}}\right),$$

**Figure 3:**
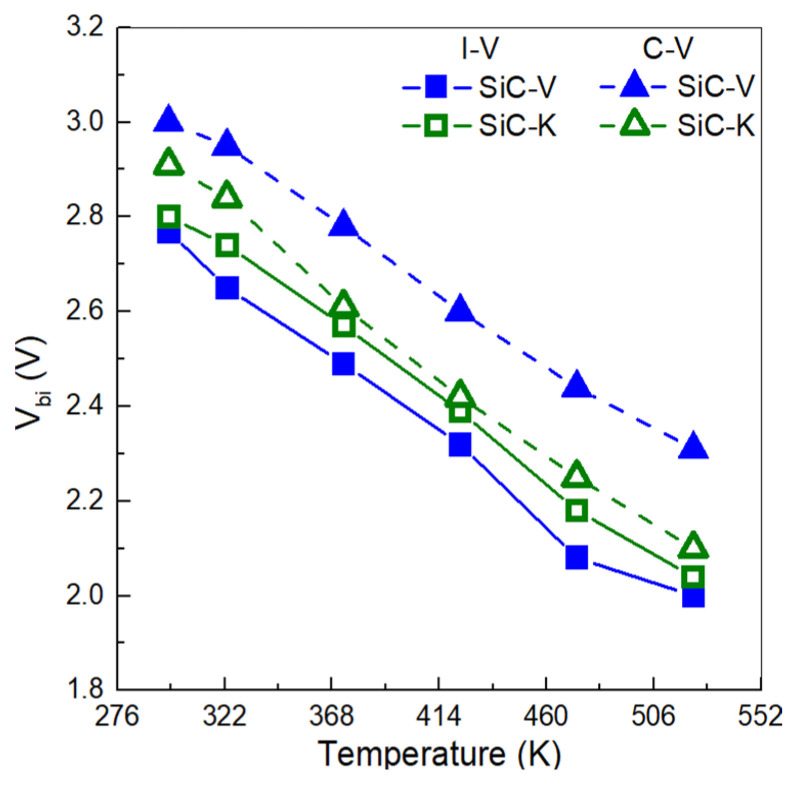
Built-in potential distributions in temperature range of 298–523 K. For the solid lines, the V_bi_ values were calculated by the C-V characteristics. For the dashed lines, the V_bi_ values were extrapolated by the forward I-V characteristics.

The V_bi_ value was calculated for both SiC-V and SiC-K. The results are reported in Figure 3. Both detectors showed a decrease in V_bi_ value with increasing temperature, with SiC-V falling from 2.77 V at 298 K to 1.90 V at 523 K. SiC-K also exhibited a decrease with increasing temperature, falling from 2.80 V to 2.05 V. This was presumably due to higher temperatures producing a narrowing of SiC bandgap, which contributed to the increase in intrinsic carrier concentration and the subsequent shift in the Fermi level [20].

Moreover, the higher V_bi_ value observed in the SiC-K diode was presumably due to a higher density of interface traps, which was induced by the combined effect of neutron energy and high temperature. This alteration can affect the electric field and enhance carrier recombination, leading to shifts in the forward J-V characteristics. A similar behaviour was observed in the study by Ze Long et al., where they investigated the polarization effect on SiC Schottky diodes before and after neutron irradiation with total fluences of 1 × 10^14^ n/cm^2^ and 7 × 10^14^ n/cm^2^. Significant changes were noted in the electrical characteristics of the detector after irradiation, particularly in the forward J-V behaviour. The conduction of the detector became problematic, with the V_F_ value exceeding 15 V due to neutron-induced damage at the metal/SiC interface [21].

The built-in voltage values can also be extrapolated from the capacitance–voltage (C-V) characteristics, yielding slightly higher V_bi_ values (see Figure 3). For example, at room temperature, the SiC-V diode exhibited a V_bi_ value of 3.0 V, which decreased to 2.38 V when the temperature was raised to 523 K.

High temperature and neutron irradiation introduced displacement damage in SiC-K, creating defects that acted as compensating centres and reduced the free carrier concentration. These defect states may have trapped carriers, altering the effective charge concentration and thereby reducing V_bi_ [18].

The difference between the built-in voltage values obtained from the I-V and C-V characteristics of the SiC diodes was due to the differing physical principles and measurement conditions of these techniques: the I-V measurements, in fact, were performed under non-equilibrium conditions, and thus recombination, injection-level effects, and parasitic resistances might have affected the observed voltage. The C-V measurements provided a more accurate built-in voltage as it reflected the equilibrium charge distribution and was less influenced by the series resistance or recombination effects [22,23].

**Figure 4:**
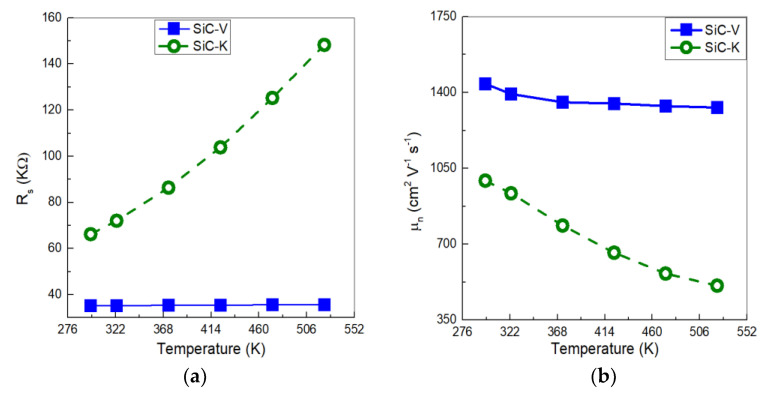
Thermal distributions. (**a**) R_S_ and (**b**) µ_n_ in the n^−^ epitaxial layer in the range of 298–523 K.

The series resistance (R_s_) of SiC-V was calculated as the slope of current density in the bias range between 3.5 and 5 V, as depicted in Figure 4a. The resulting resistance was 35.22 KΩ and remained fairly constant at varying temperatures. The electron mobility µ_n_ was instead calculated from the series resistance in the n^−^ epilayer using Equation (4):

(4) $$\mu_{n}=\frac{L}{{\mathrm{qN}}_{D}R_{s}A},$$

where L is length of the n^−^ region, N_D_ is the donor doping concentration, and A is the cross-sectional area of the diode. The µ_n_ value decreased from 1440 to 1330 cm^2^ V^−1^ s^−1^ with increasing temperature. This was due to the increase in scattering caused by the higher phonon concentration, which in turn reduced the carrier mobility. The dependence of carrier mobility with temperature for both detectors is presented in Figure 4b. It is evident how neutron irradiation significantly altered their electrical properties. This was due to the deep-level defects introduced by neutron interaction acting as acceptor levels, compensating for the shallow donor impurities in the n-type SiC epilayer. This compensation led to a reduction in free electron concentration, thereby increasing the R_s_ value to 66.22 KΩ at room temperature. Additionally, the increase in temperature up to 523 K accelerated the degradation of contacts through interdiffusion and structural changes, and, consequently, an increase in the R_s_ value to 148.33 KΩ. The combined effect of carrier concentration reduction and ON-state resistance increase caused a decrease in the electron mobility from 993 to 509 cm^2^ V^−1^ s^−1^.

H. Li et al. reported a similar decrease in the µ_n_ value as an effect of neutron irradiation by a 1 MeV China Fast Burst Reactor-II (CFBR-II) neutron source [24]. Their Schottky SiC detectors consisted of an active region formed by the contact between the n^−^ epitaxial layer (with N_D_ to 6 × 10^15^ cm^−3^ and thickness of 20 μm) and the titanium metal. At room temperature, the unirradiated detector showed µ_n_ = 860.73 cm^2^ V^−1^ s^−1^. By increasing the neutron irradiation fluence up to 6 × 10^13^ n/cm^2^, the µ_n_ value rapidly decayed to 802.02 cm^2^ V^−1^ s^−1^.

**Figure 5:**
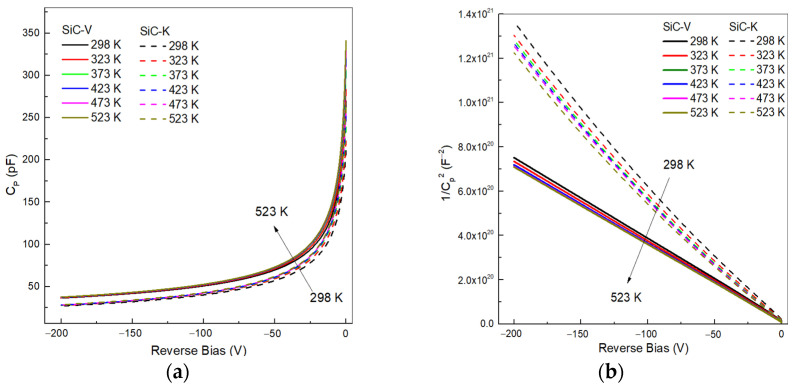
C-V characteristics of the SiC detectors in the temperature range of 298–523 K. (**a**) The C-V curve for SiC-V is reported as a solid line, while the SiC-K curve is reported as a dashed line. (**b**) Reciprocal of the squared capacitance vs. voltage of two SiC detectors.

In our experiment, the C-V measurements were performed by applying a reverse bias on two 4H-SiC p-n^−^ detectors with 250 μm-thick epilayers with an active area of 25 mm^2^ in the range of 298–523 K. At room temperature, the free carriers diffused away due to concentration gradients, leaving fixed ionized dopants at the junction, which created an internal electric field. By applying a reverse bias, the free carriers were pushed further away, enhancing the electric field and widening the depletion region. Consequently, the SiC diode reduced its ability to store charge as a direct result of the reduction in capacitance. It was observed (see Figure 5a) that higher temperatures caused an increase in the junction capacitance of the SiC-V detector. This was attributed to a slight increase in intrinsic carriers narrowing the depletion region with increasing temperature, leading to higher capacitance values [25]. After high-temperature irradiation, the C-V curves of SiC-K typically showed a reduction in capacitance at −200 V, likely due to the introduction of traps in the depletion region, which increased the resistivity and altered the effective charge distribution. A similar result was observed in the 50 µm-thick 4H-SiC p–i–n detector of A. Gsponer et al. exposed to neutron irradiation, carried out at the TRIGA MARK II reactor using a 1 MeV neutron source [26], who observed a much lower depletion capacitance after irradiating the detectors with a neutron fluence of 5 × 10^14^ n/cm^2^. In addition, they measured a constant capacitance in the bias voltage range. This was compatible with the epilayer becoming intrinsic due to the doping compensation by acceptor-type defects.

The 1/C^2^ vs. reverse bias plot showed results with a higher linearity for the unirradiated SiC-V, as shown in Figure 5b, suggesting that the doping concentration of the epitaxial layer was close to the manufacturing standard. The increase in temperature showed a negligible effect on the linearization of the C^−2^-V characteristics, while the characteristics of SiC-K showed a pronounced variation, indicating uneven dopant concentrations.

The SiC-V diode exhibited standard C-V behaviour, from which the N_D_ value at 7 × 10^13^ cm^−3^ was extracted using the linear fit of the 1/C^2^ vs. reverse bias curve. After irradiation with a neutron fluence of 5 × 10^12^ cm^−2^, the CV curves shifted downward with a decrease in N_D_ value to approximately 8% of that of the unirradiated sample.

**Figure 6:**
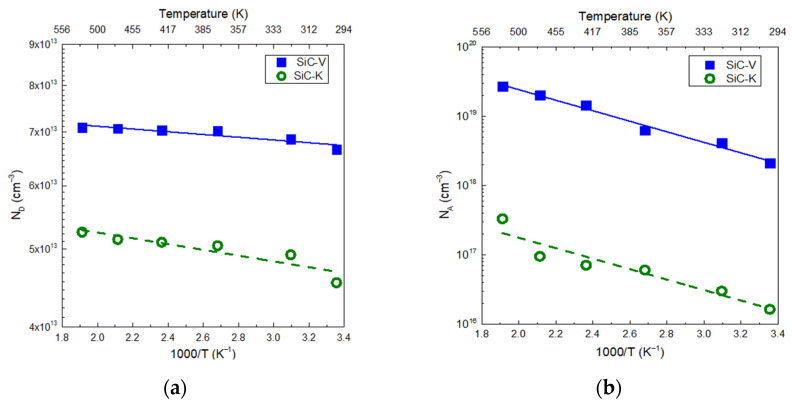
Arrhenius plots of (**a**) donor concentration for nitrogen-doped (n-type) and (**b**) acceptor concentration for aluminium-doped (p⁺-type) 4H-SiC.

Arrhenius plots of the donor and acceptor densities for the two types of p-n^−^ SiC detectors are shown in Figure 6. The N_D_ value of SiC-V was 6.7 × 10^13^ cm^−3^ at room temperature, which was close to the epitaxial doping density provided by the manufacturer. If the temperature was increased to 523 K, we observed moderate variation, raising the N_D_ value up to 7.9 × 10^13^ cm^−3^, as depicted in Figure 6a. In SiC-K, the extrapolated value of N_d_ was reduced to 4.54 × 10^13^ cm^−3^ at room temperature, which increased to 5.25 × 10^13^ cm^−3^ at 523 K. This suggested that the difference was entirely due to irradiation. The deactivation of N_D_ depends on both the energy source and fluence value [27]. Low-energy neutrons at 1 MeV caused significant displacement of Si and C atoms, forming vacancies that degrade the effective N_D_. Higher-energy neutrons at 14 MeV possibly enhanced this process by creating defect clusters rather than single defects. Additionally, as the neutron fluence increased, the cumulative defect density grew, further reducing the N_d_ value [28].

The activation energy of the N-doped epilayer in the SiC-V detector was determined from linear regression of the data in the temperature range between 298 and 523 K and was found to equal 15 ± 3 meV. After neutron irradiation, the activation energy of the N-doped epilayer in SiC-K was relatively high, at 31 ± 7 meV. The increase was likely due to the introduction of radiation-induced defects that interacted with the dopant levels and modified their ionization behaviour. Similar effects were also identified for acceptor doping density (N_A_) values in the 298–523 K temperature range. The SiC-V detector showed an N_A_ value of 2.1 × 10^18^ cm^−3^ at room temperature. If the temperature was increased up to 523 K, the calculated N_A_ value was much higher, at 2.7 × 10^19^ cm^−3^, as shown in Figure 6b. The energy required to thermally excite a hole from the acceptor level to the valence band was calculated to be 655 ± 40 meV. On the other hand, SiC-K had a much lower value of N_A_ at 1.6 × 10^16^ cm^−3^, which rose to 5.3 × 10^17^ cm^−3^ at 523 K, and a higher activation energy of 720 ± 160 meV. Several authors suggested that radiation-induced defects like carbon and silicon vacancies may capture holes, reducing the availability of free carriers and consequently increasing the apparent activation energy for ionization [29,30,31,32]. This effect could be related to the presence of defects in the SiC epilayer or in the implanted p^+^ layer, which form trap levels within the band gap, thus inducing a decrease in the N_A_ value.

### 3.2. Deep Level Transient Spectroscopy

When the pulse signal of +2.3 V was applied, the charge carriers temporarily drifted into the depletion region and returned to their previous position. However, some of these carriers were trapped by defects in the depletion region. As the temperature increased, the trapped carriers were thermally emitted, causing a change in the depletion capacitance. This variation in capacitance, known as the DLTS signal, was recorded to analyse the emission behaviours of the trapped carriers at different temperatures. Under thermal equilibrium, the emission rate (e) depended on both the trap energy level (E_t_) and the temperature, using the following Equation (5):

(5) $$\ln\left(\frac{e}{T^{2}}\right)=\ln\left(\sigma \;\beta \right)-\frac{E_{c}-E_{t}}{\mathrm{kT}},\;$$

where σ is the capture cross-section, β is approximately 3.4 × 10^21^ cm^−2^ s^−1^ in 4H−SiC [33,34], and E_c_ is the energy level of the conduction band edge ($E_{c}$).

**Figure 7:**
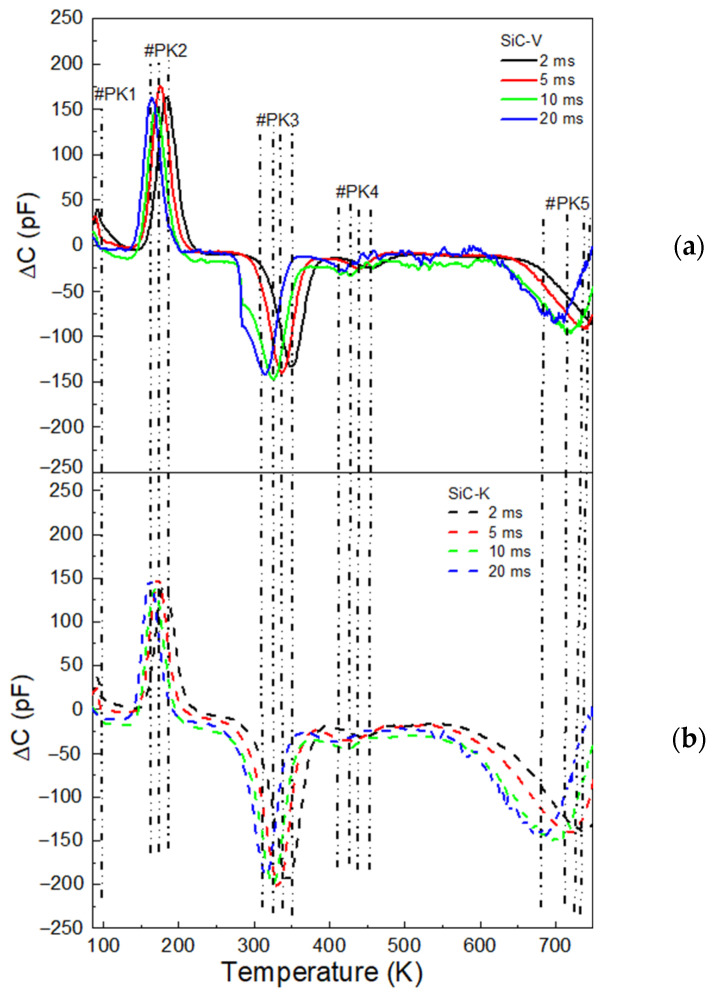
DLTS spectra. (**a**) Unirradiated SiC-V diode and (**b**) neutron-irradiated SiC-K for different delays (2, 5, 10, and 20 ms). The data were acquired with a reverse bias of −13 V and filling pulses of +2.3 V, 1 ms.

The DLTS scans revealed five peaks (PK#1–#5) in the temperature scan range of 85–750 K, which corresponded to different trap levels, as shown in Figure 7.

The first two peaks, with energy levels of 0.23 and 0.27 eV below the conduction band edge in the SiC-V device, corresponded to aluminium doping that occupied cubic and hexagonal silicon sites [34]. The presence of these peaks in the DLTS spectra resulted from the depletion of the JTE p^-^ edge structure. After SiC-K underwent neutron irradiation, the aluminium-related energy levels were only slightly affected, showing values of 0.16 eV and 0.27 eV. The trap centre related to #PK3 was found at 0.56 eV above the conduction band edge in SiC-V and could be related to the Z_1/2_ centres. Similar defect levels were observed in SiC-K, with an activation energy of 0.67 eV, which implied the existence of two distinct levels, labelled Z_1_ and Z_2_. I. Capan et al. resolved two emission lines, Z_1_ (=/0) and Z_2_ (=/0), using Laplace DLTS and assigned them to carbon vacancies (V_C_) at two different lattice sites with local cubic and hexagonal symmetry [35].

Density functional theory (DFT) predicted acceptor levels at 0.48–0.59 eV above the conduction band edge for the h-site (Z_1_) and 0.41–0.67 eV for the k-site (Z_2_) [36], in excellent agreement with our data. Electron paramagnetic resonance (EPR) provided additional evidence connecting these levels to the charge states of the V_C_ [37].

The trap level corresponding to #PK4, found at 0.84 eV below the conduction band edge, was associated with RD_1/2_ centres. These centres were believed to arise from ion implantation damage during fabrication of the SiC detectors and were generally linked to various point defects and defect complexes generated by the damage process [38]. Additionally, #PK5 (labelled EH_6/7_) was thought to be related to V_si_, isolated V_C_, or a complex defect configuration like V_C_ + V_Si_ [39]. Y. Huang et al. employed ab initio metadynamics (META) to systematically investigate the configuration space, including the direction and magnitude of bond distortion, and identified the most stable structures of V_C_. Their simulations placed V_C_ at ~1.38–1.66 eV below E_C_, in excellent agreement with EH_6/7_ [40].

Burin et al. investigated the impact of neutron irradiation on 4H-SiC detectors using Technology Computer Aided Design (TCAD) software in order to predict changes in electrical behaviour. After neutron irradiation, the simulated SiC detector exhibited an increase in the leakage current. Their simulations revealed that EH_6/7_ centres significantly affected the electrical characteristics by altering the charge collection efficiency [41].

Also, P. Gaggl et al. developed a bulk radiation damage model for TCAD simulations based on measurements of neutron-irradiated 4H-SiC diodes. Under various bias conditions, the model accurately predicted the shifting of the internal junction electric field and the degradation in charge collection efficiency [42]. They also identified EH_6/7_ centres as a major factor in carrier lifetime reduction.

The defect concentration (N_T_) was evaluated using the following Equation (6):

(6) $$\left|\Delta C\right|{=C}_{R}\frac{N_{T}\;}{N_{d}},$$

where C_R_ is the capacitance under a reverse bias of −13 V. Table 1 shows the evaluated N_T_ values and related parameters in the calculation.

In the case of Z_1/2_, the defect concentration in SiC-V was calculated as 8.06 × 10^13^ cm^−3^, which was lower than the value of 1.34 × 10^14^ cm⁻^3^ for SiC-K. The N_T_ value of RD_1/2_ was calculated as 1. 54 × 10^13^ cm^−3^ in SiC-V, which was roughly half of the 2.39 × 10^13^ cm⁻^3^ value found for SiC-K. The extrapolated $N_{T}$ value for EH_6/7_ in unirradiated SiC-V was 5.22 × 10^13^ cm⁻^3^, while the one for SiC-K (considering the effect of neutron irradiation combined with temperature) was 9.23 × 10^13^ cm⁻^3^. This result highlighted the changes in the electrical performance of SiC-K.

## 4. Conclusions

This paper reports a study of the I-V characteristics of 4H-SiC detectors at different temperatures (between 298 K and 523 K) before and after neutron irradiation with a total fluence 5 × 10^12^ n/cm^2^ at 773 K. The unirradiated detector (SiC-V) showed typical p-n junction behaviour, while the irradiated detector (SiC-K) exhibited an increased resistance and a higher ideality factor due to the neutron-induced defect generation. The effect of high temperature increased the recombination current and the resistance of the detector. The reduction in the built-in voltage (V_bi_) of the two detectors was compared, revealing that the irradiated SiC-K featured lower V_bi_ values that the unirradiated SiC-V. This difference was further deepened at high temperature due to combined effects of displacement damage and defects that reduced the free carrier concentration and electric field strength. The dopant concentrations decreased after irradiation due to defect-induced carrier compensation. Temperature increase led to partial recovery of the donor concentration (N_D_), while the N_A_ value of SiC-V remained much higher than that of SiC-K. Regarding the DLTS spectra, JTE depletion Al-related traps appeared at 0.23 eV and 0.27 eV in SiC-V, with negligible alterations following neutron irradiation at 773 K. The presence of Z_1_ and Z_2_ levels in SiC-K was possibly due to the defect level at 0.56 eV in SiC-V shifting to 0.67 eV. Also, after irradiation, a small increase in the concentration of this level was observed. RD_1/2_ defects associated with ion implantation damage due to the realization of the p^+^ anode and JTE regions were observed in SiC-K, with a trap energy of 0.84 eV. Another deep-level EH_6/7_ that was detected at around 750 K and attributed to silicon-carbon vacancy complexes increased in concentration after neutron exposure. Specifically, the Z_1/2_ defect concentration increased from 8.06 × 10^13^ cm⁻^3^ in SiC-V to 1.34 × 10^14^ cm⁻^3^ in SiC-K, while RD_1/2_ nearly doubled. Under high-temperature neutron irradiation, EH_6/7_ reached 9.23 × 10^13^ cm⁻^3^ in SiC-K.

In this work, the results indicate that high-temperature neutron irradiation significantly affects the electrical performance of SiC-K. Nevertheless, the tested 250 µm-thick 4H-SiC p-n junction detectors demonstrate promising radiation hardness and thermal stability, making them strong candidates for neutron detection in the breeding blanket of fusion power plants. Future work will focus on assessing their long-term reliability under more extreme conditions (e.g., higher temperatures, and when available, higher neutron fluences).

## Figures and Tables

**Table 1:** Trap signature of detectors and parameters used in the calculation.

Sample	Z_1/2_			RD_1/2_			EH_6/7_		
	ΔE (eV)	σ (cm^−2^)	$N_{T}$ (cm^−3^)	ΔE (eV)	σ (cm^−2^)	$N_{T}$ (cm^−3^)	ΔE (eV)	σ (cm^−2^)	$N_{T}$ (cm^−3^)
SiC-V (N_d_ = 7E13 cm^−3^; C_R_ = 121.55 pF)	0.56	5.73 × 10^−17^	8.06 × 10^13^	0.93	5.88 × 10^−15^	1.54 × 10^13^	1.69	1.53 × 10^−14^	5.22 × 10^13^
SiC-K (N_d_ = 6.7E13 cm^−3^; C_R_ = 101.68 pF)	0.67	2.72 × 10^−15^	1.34 × 10^14^	0.84	8.19 × 10^−16^	2.39 × 10^13^	1.52	2.00 × 10-^15^	9.23 × 10^13^

## Data Availability

The original contributions presented in this study are included in the article. Further inquiries can be directed to the corresponding authors.
